# Integrated web portal for non-destructive salt sensitivity detection of *Camelina sativa* seeds using fluorescent and visible light images coupled with machine learning algorithms

**DOI:** 10.3389/fpls.2023.1303429

**Published:** 2024-01-11

**Authors:** Emilio Vello, Megan Letourneau, John Aguirre, Thomas E. Bureau

**Affiliations:** Department of Biology, McGill University, Montreal, QC, Canada

**Keywords:** phenotyping, phenomics, artificial intelligence, AI, abiotic stress, salinity, *Camelina sativa*, image analysis

## Abstract

Climate change has created unprecedented stresses in the agricultural sector, driving the necessity of adapting agricultural practices and developing novel solutions to the food crisis. *Camelina sativa* (Camelina) is a recently emerging oilseed crop with high nutrient-density and economic potential. Camelina seeds are rich in essential fatty acids and contain potent antioxidants required to maintain a healthy diet. Camelina seeds are equally amenable to economic applications such as jet fuel, biodiesel and high-value industrial lubricants due to their favorable proportions of unsaturated fatty acids. High soil salinity is one of the major abiotic stresses threatening the yield and usability of such crops. A promising mitigation strategy is automated, non-destructive, image-based phenotyping to assess seed quality in the food manufacturing process. In this study, we evaluate the effectiveness of image-based phenotyping on fluorescent and visible light images to quantify and qualify Camelina seeds. We developed a user-friendly web portal called SeedML that can uncover key morpho-colorimetric features to accurately identify Camelina seeds coming from plants grown in high salt conditions using a phenomics platform equipped with fluorescent and visible light cameras. This portal may be used to enhance quality control, identify stress markers and observe yield trends relevant to the agricultural sector in a high throughput manner. Findings of this work may positively contribute to similar research in the context of the climate crisis, while supporting the implementation of new quality controls tools in the agri-food domain.

## 1 Introduction

In recent years, an ever increasing demand for land along with unprecedented environmental consequences due to climate change has significantly impacted agricultural productivity. The prevalence of saline soils is increasing worldwide due to a lack of fresh water, prolonged periods of drought and rising sea levels (Hassani et al., 2021). It is estimated that over one billion hectares (ha) of global land are currently affected by salinity, with this number increasing by two Mha per year. The issue is widespread and affects over 100 countries with severe impacts in India, China, the United States, Turkey and many other regions. For example, over 30% of land in Iran is salt-affected, leading to ongoing economic and environmental implications including decreased productivity and soil erosion, which numerous countries stand to face (Singh, 2021). Increased concentrations of sodium chloride (NaCl), lead to ionic toxicity and osmotic stress in plants. While some plants such as halophytes have the ability to tolerate salt stress, traditional crops for food use are severely impacted by NaCl, leading to inhibition of growth and low yield production (Morales et al., 2017). When coupled with other abiotic stresses such as drought, heavy metal exposure, high temperatures, and reduced humidity, these factors become limiting for crop production, leading to huge economic losses and social concerns regarding food security (Shah et al., 2018; Razzaq et al., 2021).

*Camelina sativa* (Camelina) is an undervalued oilseed crop belonging to the *Brassicaceae* family, closely related to *Arabidopsis thaliana* and other economically relevant *Brassicaceae* such as canola and the cabbage (Berti et al., 2016). This crop is native to East European/West Asian regions and was first domesticated in the late Neolithic era before being largely replaced by other competitor crops. Despite being well adapted to Canada and the northern United States due to the semi-arid, temperate and short-season climates, Camelina is not widely produced in North America (Vollmann and Eynck, 2015). It is only in more recent decades that Camelina has begun to receive a renewed interest due to its advantageous properties including low input requirements, tolerance to cold temperatures and pests and a high nutrient-density (Masella et al., 2014). Camelina seeds also contain uncommonly high levels of alpha-linolenic acid, an essential omega-3 fatty acid required for proper physical and cognitive maintenance, making it a nutritious food source (Kagale et al., 2014; Berti et al., 2016).

In recent years, there has been a surge in plant phenomics equipment and platforms, ranging from compact desktop setups to large-scale field phenotyping machines and even unmanned aerial vehicles (Vello et al., 2022; Sarkar et al., 2023). However, there is a limited availability of user-friendly tools for analyzing the vast amount of data generated by these systems, and many of the existing tools are challenging for non-computer science users to navigate (Vello et al., 2015). Furthermore, Camelina, being an emerging crop, has not been as extensively investigated as other established crops such as *Brassica napus* (canola). Our understanding of the effect of abiotic stresses such as NaCl concentration on Camelina seeds therefore remains limited (Zanetti et al., 2021). In this study, we aim to address these challenges by investigating the potential of image-based phenotyping and automated analysis through a user-friendly web portal. The SeedML portal enables the analysis of morpho-colorimetric attributes of Camelina seeds and can in turn predict their salt status. This prediction is based on image analysis and machine learning algorithms, utilizing fluorescent or visible light images acquired from a plant phenomics platform. As phenomic systems continue to innovate in response to adapting needs in the agricultural sector, the availability of accessible and powerful analysis tools will play a vital role in their success.

## 2 Materials and methods

### 2.1 Plant growth and salt treatment

**Protocol 1.** Three *Camelina sativa* (Camelina) seeds (Celine variety), were sown in 5” pots with 250 g of Sunshine mix (75-85% Canadian Sphagnum peat moss, perlite and dolomite limestone) and 450 mL of water. Plants were grown in the McGill phytotron greenhouse with a 14 h / 10 h light/dark photoperiod at a temperature of 27°C/20°C day/night. Seven days after sowing (DAS), seedlings were thinned to one per pot based on size similarity. At DAS 20, salt stress was induced through saline water treatment (final NaCl concentrations of 0, 50, 100, 150 and 200 mM), prepared using a final volume of 450 ml of water (soil water capacity). Salt treatment was progressively applied twice a day over two days. Pots were watered every day to 700g to maintain a constant NaCl concentration. Classic 20-20-20 (N-P-K) fertilizer diluted 1:10 was applied at DAS 15. Plants were randomized three times a week to avoid any positional effect in the greenhouse.

**Protocol 2.** Similar to protocol 1 but using a water capacity of 350 mL and a final weight of 600 g. Environmental temperature was set at 24°C/20°C day/night and three salt concentrations were used (NaCl at 0, 200 and 250 mM). Fertilizer was applied at DAS 8 and 15.

**Protocol 3**. Similar to protocol 2 but plants were watered twice a week without weight control and only two levels of salt concentration were used (NaCl at 0 and 200 mM). Plants were fertilized once a week.

**Protocol 4.** Similar to protocol 1 but using 200 g of soil and a 400 mL water capacity at 0 and 200mM of salt. Plants were watered to 600 g twice a week. Salt stress was induced at DAS 34.

**Plant batches:** Four different batches of plants were grown in different seasons and using different protocols in a semi-controlled environment (greenhouse) in which light and temperature may fluctuate according to the external environmental conditions. Plants in batch A were grown using protocol 1 in Spring-Summer 2018, batch B using protocol 2 in Spring 2019, batch C using protocol 3 in Fall 2020 and batch D using protocol 4 during Winter 2018.

### 2.2 Seed preparation and imaging

Harvested seeds were dried for 30 days at room temperature and then stored at 4°C. A weighing pan and an electronic balance (PB3002 DeltaRange) were used to select 0.1g or 0.05 g seeds from each plant according to the set (**Table 1**). Seeds were then transferred to petri dishes and identified with barcodes. The image acquisition was performed with the LemnaTec HTS installed at the McGill Plant Phenomics Platform (MP3, http://mp3.biol.mcgill.ca), using the visible light camera piA2400-17gc and the fluorescent light camera scA1400-17gc. Three configurations were selected; visible light top illumination (VISFRONT); visible light back illumination (VISBACK); and fluorescent illumination between 400 and 500 nm (FLUO).

**Table 1:** Seed sets and plate batches.

Set	Plate number	Seed weight x plate	Image date	Batch	Growth season	Salt concentration (mM)
1	23	0.10 g	2019-11-14	B	Spring 2019	0, 200, 250
2	35	0.10 g	2020-01-17	A	Spring-Summer 2018	0,50, 100, 150, 200
3	18	0.10 g	2021-02-25	C	Fall 2020	0, 200
4	40	0.10 g	2021-03-02	A	Spring-Summer 2018	0, 50, 100, 150, 200
5	10	0.05 g	2021-03-02	A	Spring-Summer 2018	0, 200
6	15	0.10 g	2021-04-27	D	Winter 2018	0, 200
M	18	0.10 g	2020-01-22	A/B	A and B mixed	0, 200

### 2.3 Software development

The three main components of the web portal software (the web interface, the image analysis and the machine learning implementation), were implemented on Java OpenJDK 17 + 35 and Apache Tomcat 10.1.10. The web portal was developed using JSP, HTML, JavaScript, CSS. The image analysis and machine learning modules were developed using ImageJ 1.53a (Schneider et al., 2012), Fiji (Schindelin et al., 2012) and weka 3.9.4 (Frank et al., 2016), respectively as main packages and Java as programming language. An adapted version of the “combined contour tracing and region labeling” proposed by Burger and Burge (2008, 2016) was implemented as part of the segmentation algorithm. SeedML was assigned as the name of the portal.

### 2.4 SeedML web portal

The web portal runs on a Dell R910 server with 512 GB of RAM and two MD1200 storage devices 72 TB at McGill University. The SeedML web portal is accessible through the internet address https://sites.google.com/view/seedml or http://mp3.biol.mcgill.ca/seedml. The prediction of the salt status analysis is performed in the following steps. 1) Seed detection setup; 2) Training images; 3) Testing images; 4) Process; 5) Phenotypic traits; 6) Seed classification. The portal could also be used to analyze morpho-colorimetric traits alone. In this case, steps 1, 3, 4 and 5 are required.

### 2.5 Seed detection setup

In this step, the user can select different thresholds for some image properties or the application of determined algorithms in order to set up the segmentation parameters, seed and background identification. It is possible to set the scale of pixels per centimeter assuming a pixel aspect ratio of one. The segmentation parameters are easily set up by clicking or dragging and dropping a sample image of a plate on the box under the title “original image”. After clicking the refresh button, the processed images on the right box will give a preview of some intermediary (pre-processed) and final results of the segmentation. The adjustment and refreshing of the segmentation parameters is performed until the identification of the seeds is archived. This configuration can be downloaded to the local disk to be reused in future analysis. The portal has three pre-set configurations used for this article, visible light top illumination, visible light back illumination and fluorescent light.

### 2.6 Training images

One or more images for each growth condition (salt and normal) are uploaded by clicking or dragging and dropping to the respective panel. These images are used to train the different machine learning algorithms. The garbage icon allows the user to clean up the content of the panel. The uploading operation is successfully achieved when a scaled image and its names are shown in the corresponding list.

### 2.7 Testing images

The center panel is designed to upload the images of the seed plates to be analyzed by dragging and dropping or clicking. This section is also used if a morpho-colorimetric analysis only is desired. Before moving to the next step, the user has to wait until a small-scale copy of each image is shown in the center panel.

### 2.8 Image analysis and classification process

Once the training and testing images are uploaded, the user can run the process of image analysis and classification using the start button. The classification process can be based on all, only morpho or only color attributes (**Tables 2**, **3** respectively). The button in the middle panel allows the user to change the option. Once the process is complete, the third panel central label will change from “X” to “✓”.

**Table 2:** Description of morphological features.

Identification	Definitions
Area	Number of pixels representing the seed in the image. (Joly-Lopez et al., 2017)
Perimeter	Length of the outer contour of the pixels representing the seed in the image. (Joly-Lopez et al., 2017)
Circularity	Ratio between the circumference square and the area. (Camargo et al., 2014)
Compactness	Ratio between the area and the perimeter (Burger and Burge, 2008, 2016).
Major axis	Axis where a physical body requires less effort to rotate. It extends from the centroid (center of gravity) to the widest part of the object (Burger and Burge, 2008, 2016), in this case the pixels presenting the seed in the image.
Minor axis	Axis perpendicular to the major axis.
Eccentricity	Ratio between the major axis and the minor axis of the digital plant (Burger and Burge, 2008, 2016). The minor axis extends from the centroid to the narrowest part perpendicular to the major axis.

**Table 3:** Description of colorimetric features.

Identification	Description
Grey intensity peak (hisgreypeak)	Intensity value having the bigger frequency from the pixels representing the seed. It is the higher peak of the intensity value histogram. (Joly-Lopez et al., 2017)
Q_1_ grey pixels (q1grey)	First quartile of the pixel grey value distribution. (R+G+B)/3.
Q_2_ grey pixels (q2grey)	Second quartile of the pixel grey value distribution. (R+G+B)/3.
Q_3_ grey pixels (q3grey)	Third quartile of the pixel grey value distribution. (R+G+B)/3.
Q_1_ red channel pixels (q1r)	First quartile of the pixel red channel value distribution.
Q_2_ red channel pixels (q2r)	Second quartile of the pixel red channel value distribution.
Q_3_ red channel pixels (q3r)	Third quartile of the pixel red channel value distribution.
Q_1_ green channel pixels (q1g)	First quartile of the pixel green channel value distribution.
Q_2_ green channel pixels (q2g)	Second quartile of the pixel green channel value distribution.
Q_3_ green channel pixels (q3g)	Third quartile of the pixel green channel value distribution.
Q_1_ blue channel pixels (q1b)	First quartile of the pixel blue channel value distribution.
Q_2_ blue channel pixels (q2b)	Second quartile of the pixel blue channel value distribution.
Q_3_ blue channel pixels (q3b)	Third quartile of the pixel blue channel value distribution.
Higher 16 color class (hue16max)	Color class having the higher number of pixels from a hue channel 16 class pixel division in the HSB color space.
Higher 32 color class (hue32max)	Color class having the higher number of pixels from a hue channel 32 class pixel division in the HSB color space.
Higher 64 color class (hue64max)	Color class having the higher number of pixels from a hue channel 64 class pixel division in the HSB color space.

### 2.9 Phenotypic traits

A summary table with the seed count and the average seed size, seed length, seed width and seed circularity per plate is shown. If the pixels/metric scale is set up, the metric attributes are displayed in millimeters. Clicking on the image name, a new web page is presented with the object (seed) research region, the original objects (seeds), the color classification, the false color representation and a table with selected morpho-colorimetric attributes per seed (Joly-Lopez et al., 2017; Vello et al., 2022). Each seed can be traced into the image using the ID attribute of the table in the “original objects” image. Most of the table can be downloaded in a comma-separated values (CSV) file format supported by a large variety of software such as Microsoft Excel, Google Sheets, LibreOffice, R.

### 2.10 Seed classification

**Figure 1:**
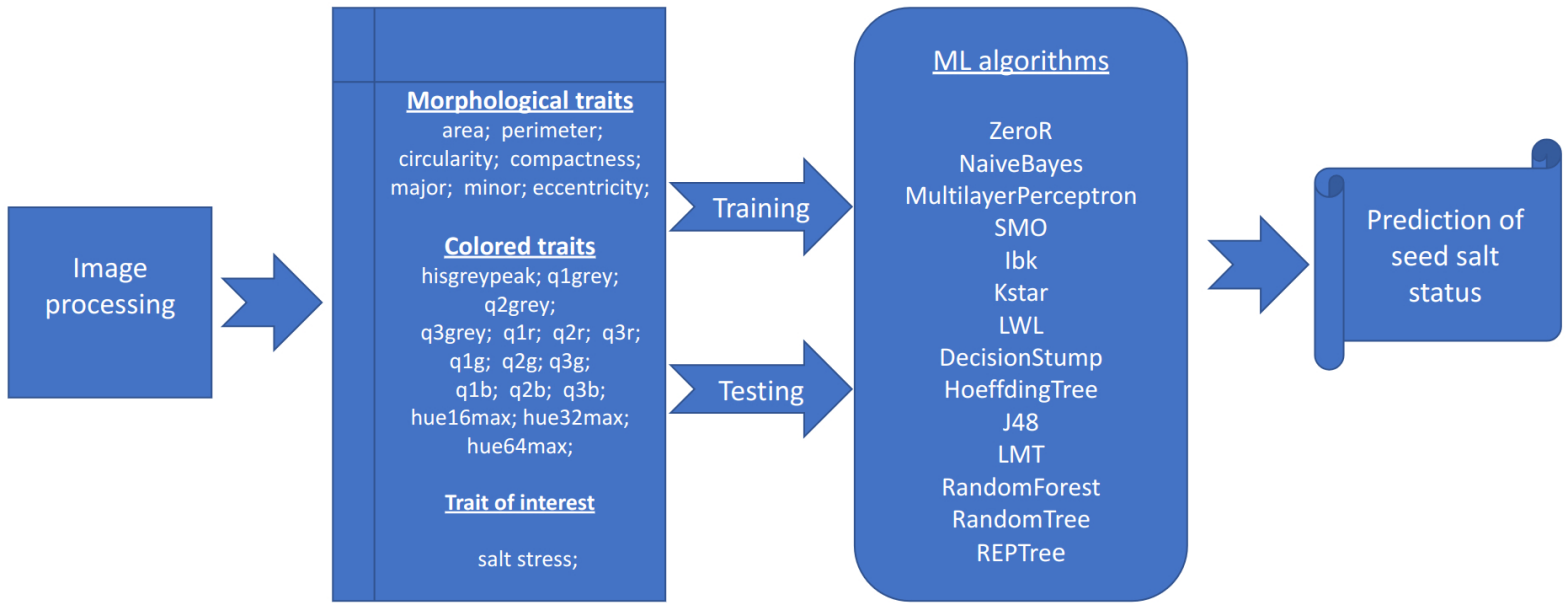
Image and data analysis pipeline. Graphical representation of the analysis pipeline implemented in the SeedML portal for plants grown under normal or salt stress conditions.

The salt status of each plate is determined by the average of the percentage of salt/non-salt among all algorithms **Table 4** included in the portal (**Figure 1**). If the percentage is greater than 50, then the plate is marked with the stress status. This section of the software displays a table containing the individual percentages for each algorithm and the predicted status of the plate. As described in “phenotypic traits”, the details of the plate can be obtained by clicking on its name.

**Table 4:** Description of the machine learning algorithms.

Classifier	Description	Reference weka packages
ZeroR	A rule algorithm that predicts the majority class in case of normal data or the average value.	https://weka.sourceforge.io/doc.dev/weka/classifiers/rules/ZeroR.html
NaiveBayes	Implements a standard probabilistic naive Bayes algorithm using estimator classes.	https://weka.sourceforge.io/doc.dev/weka/classifiers/bayes/NaiveBayes.html
MultilayerPerceptron	Implements a type of artificial neural network algorithm which can be expressed as standard mathematical functions.	https://weka.sourceforge.io/doc.dev/weka/classifiers/functions/MultilayerPerceptron.html
SMO	Sequential minimal optimization. This class implements a support vector classification that can be expressed as standard mathematical functions.	https://weka.sourceforge.io/doc.dev/weka/classifiers/functions/SMO.html
IBk (lbk)	Implements a k-nearest-neighbour classification algorithm.	https://weka.sourceforge.io/doc.dev/weka/classifiers/lazy/IBk.html
Kstar	Implements the nearest neighbour algorithm with a generalized distance function.	https://weka.sourceforge.io/doc.dev/weka/classifiers/lazy/KStar.html
LWL	Implements a general algorithm for locally weighted learning.	https://weka.sourceforge.io/doc.dev/weka/classifiers/lazy/LWL.html
DecisionStump	Implements a decision tree using only one level for splitting.	https://weka.sourceforge.io/doc.dev/weka/classifiers/trees/DecisionStump.html
HoeffdingTree	Implements a Hoeffding tree algorithm.	https://weka.sourceforge.io/doc.dev/weka/classifiers/trees/HoeffdingTree.html
J48	Implements a C4.5 decision tree learner algorithm.	https://weka.sourceforge.io/doc.dev/weka/classifiers/trees/J48.html
LMT	Logistic model trees. It builds classification trees with regression functions at their leaves.	https://weka.sourceforge.io/doc.dev/weka/classifiers/trees/LMT.html
RandomForest	Implements the algorithm for building a forest of random trees.	https://weka.sourceforge.io/doc.dev/weka/classifiers/trees/RandomForest.html
RandomTree	Given a number of random features for each node, this class builds a tree without pruning.	https://weka.sourceforge.io/doc.dev/weka/classifiers/trees/RandomTree.html
REPTree	Implements a fast tree learning that reduces the error pruning.	https://weka.sourceforge.io/doc.dev/weka/classifiers/trees/REPTree.html

(Witten et al., 2011; Smith and Frank, 2016).

### 2.11 Testing procedure

All the output data shown in this work has been processed using the SeedML portal in order to assess its power to identify morpho-colorimetric features of seeds and predict the salt status of the plates. The exception is the performance of the machine learning algorithms that has been done before the portal implementation. After uploading and processing the images into the portal, the morpho-colorimetric features were downloaded using the phenotypic traits option and plotted in R. The prediction tests were divided into two groups: inside sets and between sets. For inside sets, three tests for each camera (FLUO: fluorescent, VISFRONT: visible top light, VISBACK: visible back light), attribute (all, only morpho, only color), set (1-6) and salt concentration (50 mM, 100 mM, 150 mM, 200 mM, and 250 mM) were performed (**Supplementary Table 1**). For between sets, the k-fold cross-validation method with k=10 (Sakeef et al., 2023) was used on 200 mM only since this concentration is present in all sets. The k-fold cross-validation prevents underfitting or overfitting of the model, aligning with the sample size and the split between testing and training in the various tests (Saharan et al., 2021; Charilaou and Battat, 2022; Prusty et al., 2022). The portal has been tested in Firefox and QuteBrowser.

### 2.12 Evaluation of the prediction process

The performance and effectiveness of the prediction status of seeds and plates is measured using five metrics commonly used in benchmarks of machine learning algorithms: accuracy (Equation 1), sensitivity Equation 2, specificity Equation 3, precision Equation 4 and F1 score Equation 5 (Xu et al., 2022; Yang et al., 2023).


(1) $$Accuracy\;=\frac{\Sigma \left(true\;positives\right)\;+\;\Sigma \left(true\;negatives\right)}{total}$$

(2) $$\;Sensitivity\;=\;\frac{\Sigma \left(true\;positive\right)}{\Sigma \left(true\;positive\right)\;+\;\Sigma \left(false\;negative\right)}$$

(3) $$Specificity\;=\;\frac{\Sigma \left(true\;negatives\right)}{\Sigma \left(true\;negatives\right)\;+\;\Sigma \left(false\;positives\right)}$$

(4) $$Precision\;=\;\frac{\Sigma \left(true\;positives\right)}{\Sigma \left(true\;positives\right)\;+\;\Sigma \left(false\;positives\right)}$$

(5) $$F1\;Score\;=\;\frac{2\;\times \;Precision\;\times \;Sensitivity}{Precision\;\times \;Sensitivity}$$

### 2.13 Portal availability

The SeedML portal can be accessed at https://sites.google.com/view/seedml, where images, additional information, and access to the portal, including current and future mirrors, can be found. Alternatively, it is possible to access it directly at http://mp3.biol.mcgill.ca/seedml. For any inquiries or issues, including mirror installations, please contact the corresponding authors.

## 3 Results

### 3.1 Morpho-colorimetric features under normal and salt conditions

**Figure 2:**
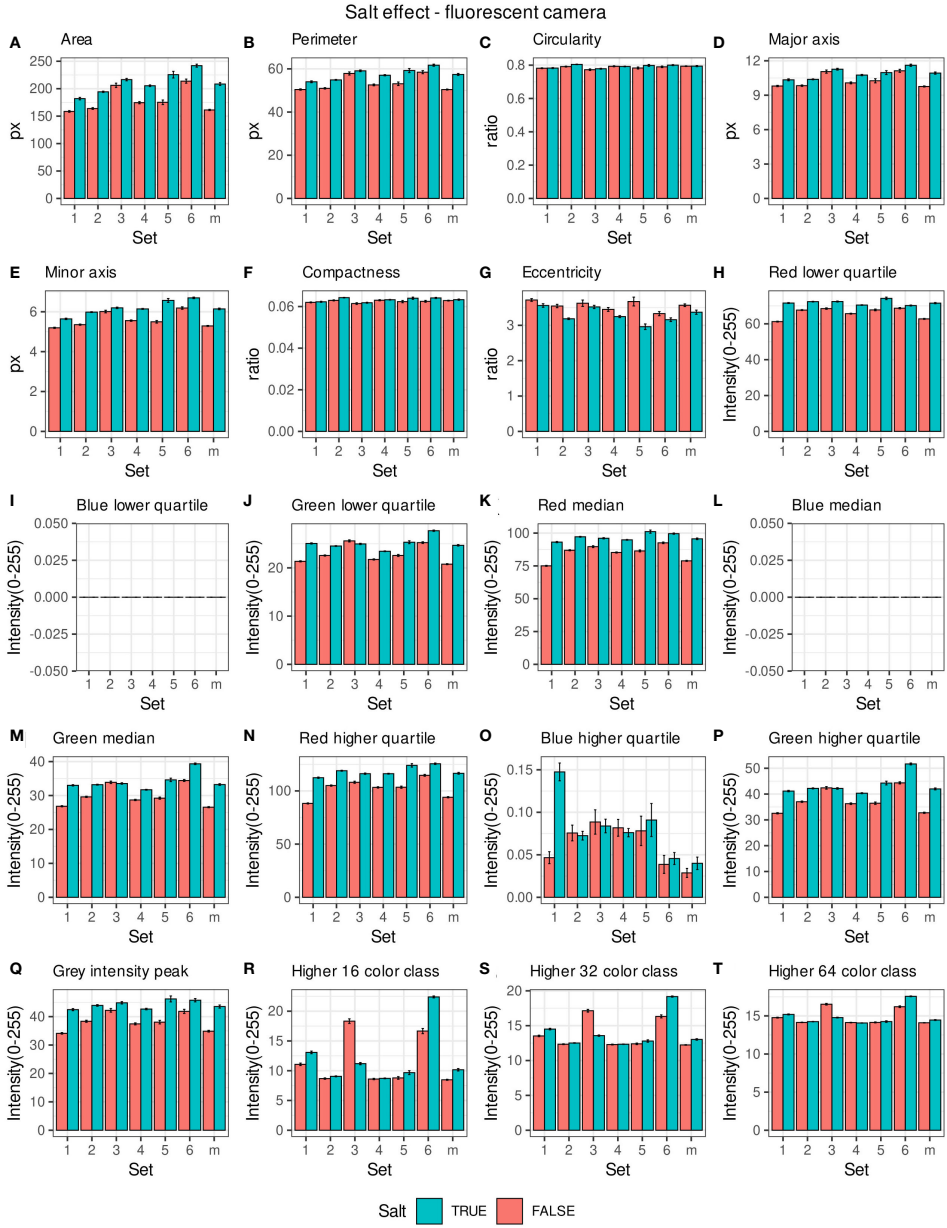
Morpho-colorimetric features from the back light visible light camera. Means and SEMs of the morpho-colorimetric features under normal and salt conditions for the 6 sets as well as the mix set (m). **(A)** Area, **(B)** Perimeter, **(C)** Circularity, **(D)** Major axis, **(E)** Minor axis, **(F)** Compactness, **(G)** Eccentricity, **(H)** Red lower quartile, **(I)** Blue lower quartile, **(J)** Green lower quartile, **(K)** Red median, **(L)** Blue median, **(M)** Green median, **(N)** Red higher quartile, **(O)** Blue higher quartile, **(P)** Green higher quartile, **(Q)** Grey Intensity peak, **(R)** Higher 16 color class, **(S)** Higher 32 color class, **(T)** Higher 64 color class.

**Figure 3:**
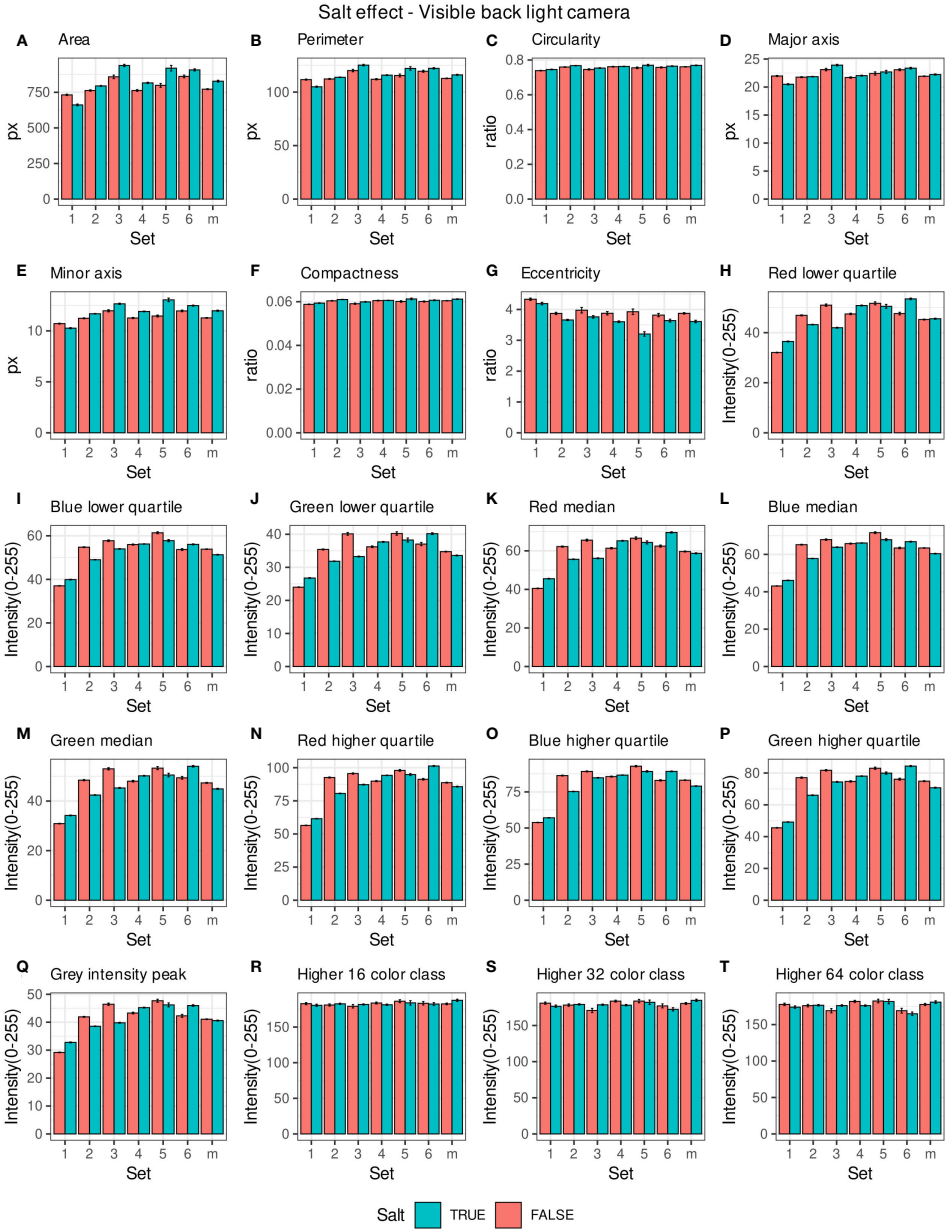
Morpho-colorimetric features from the back light visible light camera. Means and SEMs of the morpho-colorimetric features under normal and salt conditions for the 6 sets as well as the mix set (m). **(A)** Area, **(B)** Perimeter, **(C)** Circularity, **(D)** Major axis, **(E)** Minor axis, **(F)** Compactness, **(G)** Eccentricity, **(H)** Red lower quartile, **(I)** Blue lower quartile, **(J)** Green lower quartile, **(K)** Red median, **(L)** Blue median, **(M)** Green median, **(N)** Red higher quartile, **(O)** Blue higher quartile, **(P)** Green higher quartile, **(Q)** Grey Intensity peak, **(R)** Higher 16 color class, **(S)** Higher 32 color class, **(T)** Higher 64 color class.

Morpho-colorimetric seed features were compared between any concentration of salt and non-salt growing conditions under visible back light (VISBACK) and fluorescent light cameras (FLUO). The area, perimeter, major and minor axis have shown higher values in the salt group under FLUO (**Figure 2**). However, this pattern was not observed in the VISBACK (**Figure 3**). In both cameras, the eccentricity has shown higher values in the non-salt group among all the sets. The color related features in the VISBACK have not presented defined patterns among the sets. For example, the red lower quartile feature in the non-salt group is lower in set number 1 and higher in set number 3. The grey intensity peak non-salt value is higher in set number 2 but it is lower in sets 4 and 6. In the case of FLUO, a pattern was found in some of the color-related features. This is the case in the red lower, median and higher quartiles where the salt group has shown higher values. Almost no signal was observed from the blue channel. This was expected as the fluorescent information is represented in the red channel under FLUO.

The values of the area in non-salt condition groups are approximately 150 px for sets 1, 2, 4, 5 and M and slightly higher than 200 px for sets 3 and 6 (**Figure 2A**), under FLUO. This pattern is observed for the perimeter, major and minor axis as well (**Figures 2B, D, E**). The sets 2, 4 and 5 come from batch A and set 1 from batch B. The M set is a mix of A and B. Set 3 and 6 are taken from batch C and D respectively. In the VISBACK images, the area values for sets 3 and 6 are slightly higher than the other batches. The non-area related features, circularity, compactness and eccentricity show the same patterns among the sets under the VISBACK and FLUO as expected (**Figures 2C, F, G**, **3C, F, G**).

### 3.2 Pixel to metric conversion agreement and seed count

The conversion from pixels to metrics was done using the inside diameter of the petri dish plate at 8.50 cm. The diameter of the plate under the FLUO is 846.50 pixels (px) giving 99.58 px/cm (**Supplementary Figure 1A**). The same diameter under the VISBACK is 1812 px giving 213.17 px/cm (**Supplementary Figure 1B**). The double of the major and the minor axes can be used as a proxy to the length and width respectively. The average major and minor axes in the FLUO are the 9.76 px and 5.17 px giving a length of 1.95 mm and a width of 1.03 mm. In the case of the VISBACK, the averages are 21.75 px and 10.83 px giving a length of 2 mm and a width of 1 mm. Our manual calculation using a ruler on the actual seeds (**Supplementary Figure 1C**), has shown a length of 2 mm. The automatic seed count from the images having 0.10 g/seeds per plate revealed that the average number of seeds is 92, (95% CI [89.53, 95.20]) for FLUO, 84, (95% CI [80.46, 88.25]) for VISFRONT and 95, (95% CI [92.54, 99.31]) for VISBACK (**Supplementary Figure 1D**).

### 3.3 Performance of machine learning algorithms in individual seeds

**Figure 4:**
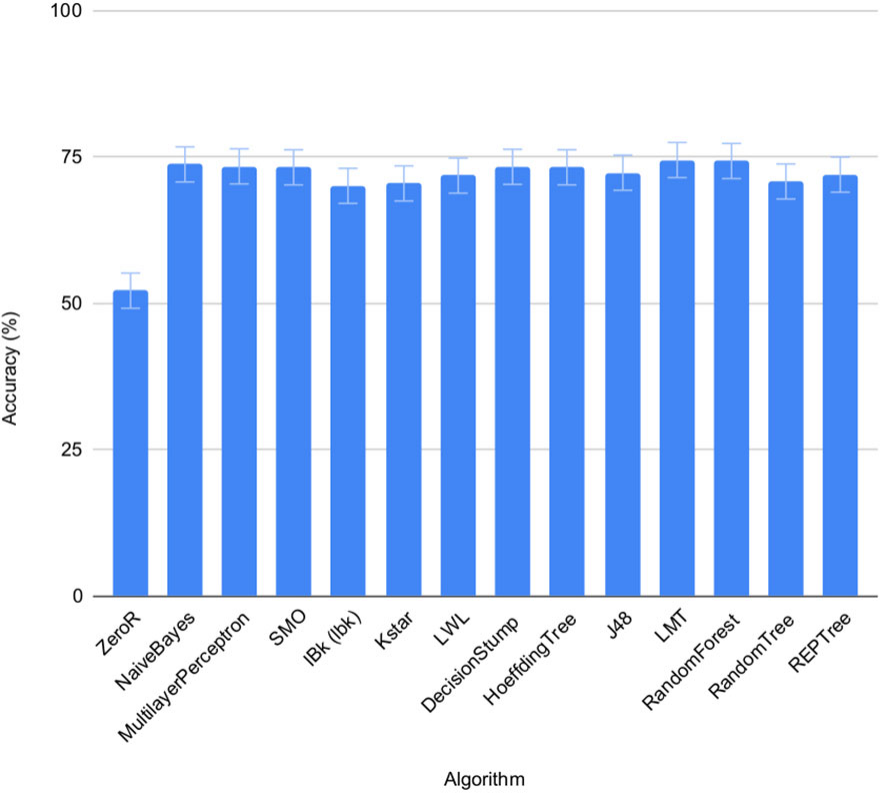
Performance of machine learning algorithms on individual seeds. Mean accuracy and SEMs for selected machine learning algorithms in set 1 and 2 under normal and salt conditions shown together.

The accuracy of the 13 pre-selected machine learning algorithms from the WEKA package (Frank et al., 2016) to predict salt status of the seeds was tested using set 1 and 2 on individual seeds. FLUO and VISBACK images were computed all together (**Figure 4**), using one or two plates as training for each condition. The ZeroR showed an accuracy of 52%, NaiveBayes 74%, MultilayerPerceptron 73%, SMO 73%, IBk 70%, Kstar 71%, LWL 72%, DecisionStump 73%, HoeffdingTree 73%, J48 72%, LMT 75%, RandomForest 74%, RandomTree 71% and REPTree 72%. The ZeroR algorithm was not implemented in the portal because of its low accuracy compared to the rest of the algorithms.

### 3.4 Portal performance inside sets using 0 and 200 mM (0-200mM) salt concentrations

The performance of the portal was evaluated within various sets, specifically focusing on salt concentrations of 0 mM and 200 mM (0-200mM). This assessment encompassed both the predictive capabilities of the portal and the type of camera used (fluorescent and visible light), across different groups. Each concentration of salt and non-salt plates was subjected to triplicate testing. During the training phase, either one or three plates were employed, depending on the specific test. The majority of tests were conducted with just one training plate per group, which represents the minimum information necessary for the classification algorithms.

**Figure 5:**
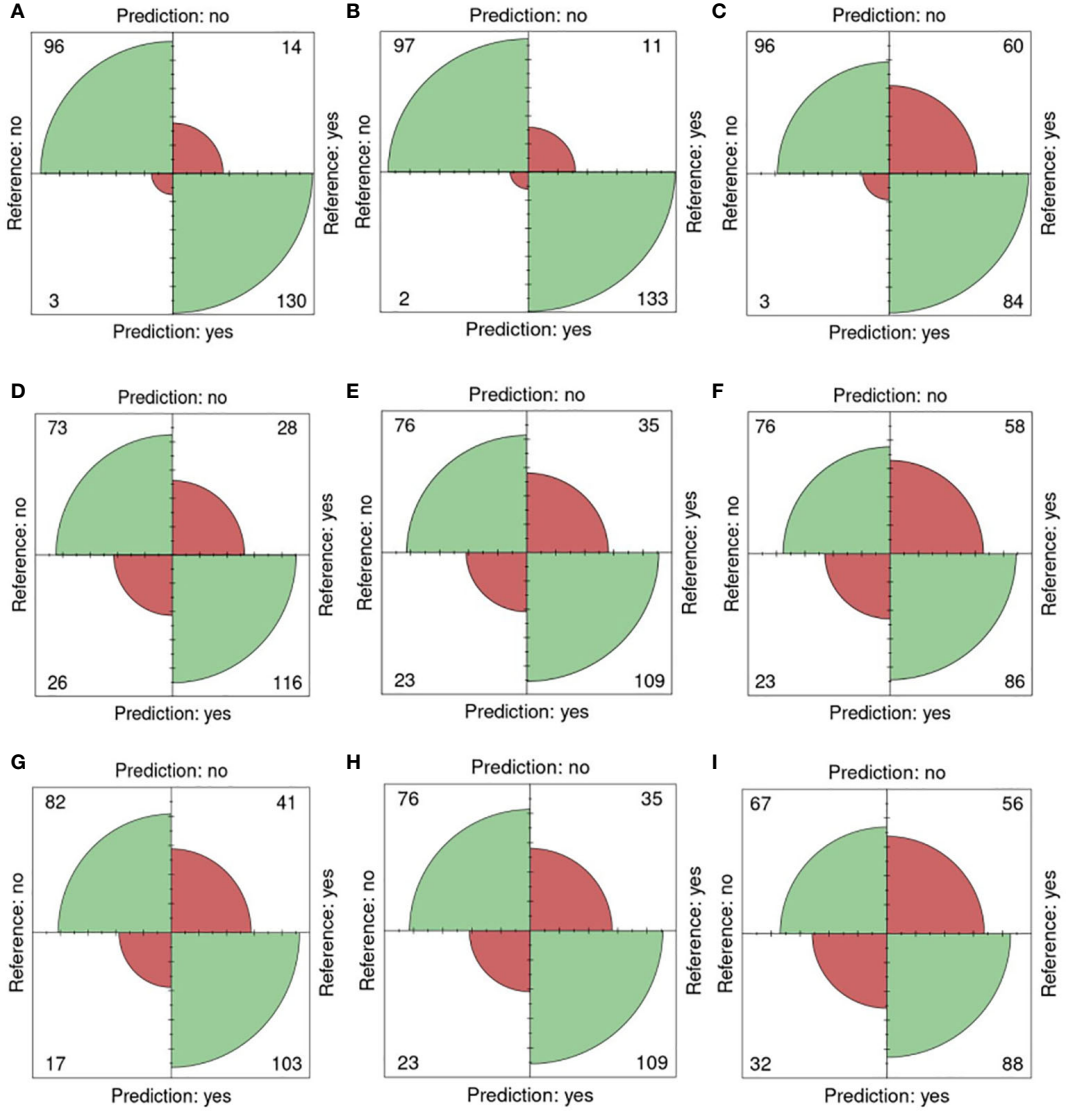
Confusion matrices for 0 and 200mM. Reference (real) versus prediction plots for sets 1 through 6 using one training plate for each condition (salt/ non-salt) and set with n=3 for. **(A)** FLUO, all attributes. **(B)** FLUO, color attributes. **(C)** FLUO, morphological attributes. **(D)** VISBACK, all attributes. **(E)** VISBACK, color attributes. **(F)** VISBACK, morphological attributes. **(G)** VISFRONT, all attributes. **(H)** VISFRONT, color attributes. **(I)** VISFRONT, morphological attributes. (FLUO, Fluorescent images; VISBACK, visible back light images; VISFRONT, visible top light images).

In **Figure 5**, confusion matrices for the 0-200 mM salt concentrations, utilizing one training plate for each group, are presented. Among the 243 plates analyzed, 96 plates were accurately classified as non-salt, and 130 were correctly identified as salt (**Figure 5A**). Only 3 were incorrectly classified as salt, and 14 were misclassified as non-salt when using fluorescent images (FLUO) with all attributes.

When only color attributes were considered, 2 plates were wrongly classified as non-salt, and 11 were misclassified as salt. However, 97 plates were accurately categorized as non-salt, and 133 were correctly identified as salt (**Figure 5B**). The classification of plates using solely morphological attributes resulted in 3 misclassified plates and 96 correctly classified as non-salt. However, 60 plates were wrongly classified as non-salt, but 84 were correctly identified as salt (**Figure 5C**). For visible back light (VISBACK) with all attributes, the portal incorrectly grouped 26 plates as salt and 28 plates as non-salt. Nonetheless, 73 plates were accurately categorized as non-salt, and 116 were correctly identified as salt (**Figure 5D**).

In the color and morphological features of VISBACK images (**Figures 5E, F**), 76 plates were correctly classified, and 23 were misclassified as non-salt. Notably, 109 plates exhibited accurate salt classification when considering color attributes, surpassing the 86 plates correctly classified using morphological attributes. Conversely, there were 35 instances of misclassification for color attributes and 58 for morphological attributes. The classification of VISFRONT images was similar in the number of plates to that of VISBACK. However, when considering all attributes, VISFRONT achieved higher accuracy in classifying 5 more plates as non-salt but was 13 plates less accurate in classifying salt content. The classification results were identical to VISBACK when using only color attributes. In the case of morphological attributes, VISFRONT outperformed VISBACK by accurately classifying 2 more plates as salt but underperformed by 9 plates in the non-salt classification (**Figures 5G–I**).

**Table 5** provides an overview of the five selected metrics employed to evaluate the portal’s performance across all sets, using a concentration level of 0 and 200 mM (0-200nM). When utilizing just one training plate, the FLUO analysis achieved impressive results, with an accuracy of 0.93, a sensitivity of 0.90, a specificity of 0.96, a precision of 0.97, and an F1 score of 0.93 across all attributes. In comparison, the color feature subset yielded slightly higher results, with an accuracy of 0.94, a sensitivity of 0.92, a specificity of 0.97, a precision of 0.98, and an F1 score of 0.95. On the other hand, the morphological subset exhibited metrics of 0.74, 0.58, 0.96, 0.96, and 0.72, respectively.

**Table 5:** Performance descriptors within groups in 0 versus 200mM.

Set	Camera	Attributes	Training Plates*	Accuracy Equation 1	Sensitivity Equation 2	Specificity Equation 3	Precision Equation 4	F1 score Equation 5
1-6	Fluo	All	1	0.93	0.90	0.96	0.97	0.93
1-6	Fluo	Color	1	0.94	0.92	0.97	0.98	0.95
1-6	Fluo	Morpho	1	0.74	0.58	0.96	0.96	0.72
1-6	VisBack	All	1	0.77	0.80	0.73	0.81	0.81
1-6	VisBack	Color	1	0.76	0.77	0.77	0.83	0.79
1-6	VisBack	Morpho	1	0.67	0.60	0.77	0.79	0.60
1-6	VisFront	All	1	0.76	0.72	0.83	0.86	0.78
1-6	VisFront	Color	1	0.76	0.76	0.77	0.83	0.79
1-6	VisFront	Morpho	1	0.64	0.61	0.68	0.73	0.67
1-6	Fluo	All	3	0.98	0.97	1	1	0.98
1	Fluo	All	3	0.97	0.93	1	1	0.96
2	Fluo	All	3	1	1	1	1	1
3	Fluo	All	3	0.98	0.97	1	1	0.98
4	Fluo	All	3	1	1	1	1	1
5	Fluo	All	3	1	1	1	1	1
6	Fluo	All	3	0.96	0.96	1	1	0.98

* Training plates per condition group. (Fisher’s exact test p<0.01 for all cases). Fluo, fluorescent images; VisBack, Visible back light images; VisFront, Visible top light images.

For VISBACK with all attributes, the system achieved an accuracy of 0.77, a sensitivity of 0.80, a specificity of 0.73, a precision of 0.81, and an F1 score of 0.81. In contrast, the color and morphological tests generated results of 0.76, 0.77, 0.77, 0.83, and 0.79, as well as 0.67, 0.60, 0.77, 0.79, and 0.60, respectively. When assessing VISFRONT, considering all attributes, an accuracy of 0.76, a sensitivity of 0.72, a specificity of 0.83, a precision of 0.86, and an F1 score of 0.78 were achieved. Using only the color attributes, the results were 0.76, 0.76, 0.77, 0.83 and 0.79. Meanwhile, employing only the morphological attributes yielded scores of 0.64, 0.61, 0.68, 0.73 and 0.67, respectively.

These findings suggest that the FLUO analysis outperforms VISBACK, and in turn, VISBACK outperforms VISFRONT. Moreover, it becomes evident that color attributes exhibit greater effectiveness than morphological attributes in accurately predicting the salt status of seeds in plates.

When three training plates were used in FLUO (as presented in **Table 5**), the five metrics consistently demonstrated values ranging from 0.96 to 1, whether considered across all sets collectively or individually. The lowest recorded value, which was 0.96, occurred in accuracy and sensitivity for set 6, and in the F1 score for set 1. These results indicate a near 100% effectiveness in detection.

### 3.5 Portal performance inside sets using other salt concentrations

To evaluate the performance of the portal and the type of camera (fluorescent or visible light) across various concentrations, sets 2 and 4 were tested using 50 mM, 100 mM and 150 mM in addition to 200 mM of salt versus non-salt under both fluorescent (FLUO) and visible backlight (VISBACK) images. The performance metrics are presented in **Tables 6** and **7**.

**Table 6:** Performance descriptors within groups in set 2 and 4 using one training plate for each condition group under fluorescent light images (FLUO).

Concentration	Attributes	Accuracy Equation 1	Sensitivity Equation 2	Specificity Equation 3	Precision Equation 4	F1 score Equation 5	p-value*
0-200nM	All	0.95	0.90	1	1	0.95	< 2.2e-16
0-150mM	All	0.72	0.61	0.80	0.73	0.67	1.815e-4
0-100mM	All	0.77	0.51	1	1	0.67	4.257e-06
0-50mM	All	0.64	0.75	0.54	0.59	0.65	0.01098
0-200mM	Color	0.94	0.88	1	1	0.93	< 2.2e-16
0-150mM	Color	0.80	0.71	0.88	0.83	0.77	9.294e-08
0-100mM	Color	0.75	0.48	1	1	0.66	4.551e-08
0-50mM	Color	0.56	0.50	0.61	0.52	0.51	0.3616
0-200mM	Morpho	0.94	0.88	1	1	0.88	< 2e-16
0-150mM	Morpho	0.74	0.86	0.64	0.67	0.75	7.432e-06
0-100mM	Morpho	0.74	0.58	0.88	0.82	0.69	1.498e-05
0-50mM	Morpho	0.53	0.94	0.19	0.50	0.65	0.09707

* Fisher’s exact test.

**Table 7:** Performance descriptors within groups in set 2 and 4 using one training plate for each condition group under visible back light images (VISBACK).

Concentration	Attributes	Accuracy Equation 1	Sensitivity Equation 2	Specificity Equation 3	Precision Equation 4	F1 score Equation 5	p-value*
0-200mM	All	0.71	0.90	0.52	0.66	0.76	3.601e-05
0-150mM	All	0.73	0.67	0.79	0.73	0.70	7.798e-05
0-100mM	All	0.47	0.56	0.38	0.46	0.50	0.6561
0-50mM	All	0.42	0.63	0.23	0.42	0.51	0.3616
0-200mM	Color	0.74	0.69	0.78	0.76	0.72	7.328e-06
0-150mM	Color	0.73	0.77	0.69	0.68	0.78	4.174e-05
0-100mM	Color	0.49	0.52	0.43	0.48	0.51	1
0-50mM	Color	0.60	0.83	0.40	0.55	0.66	0.0264
0-200mM	Morpho	0.82	0.64	1	1	0.78	2.628e-11
0-150mM	Morpho	0.60	0.36	0.80	0.61	0.46	0.1251
0-100mM	Morpho	0.61	0.36	0.36	0.86	0.47	0.03802
0-50mM	Morpho	0.44	0.44	0.45	0.41	0.42	0.4959

* Fisher’s exact test.

For the 0-200 mM concentrations, employing all attributes resulted in an accuracy of 0.95, a sensitivity of 0.90, a specificity and precision of 1, and an F1 score of 0.95, with a Fisher’s exact test p-value lower than 2.2e-16. When testing at 0-150 mM, the metrics displayed an accuracy of 0.72, a sensitivity of 0.61, a specificity of 0.80, a precision of 0.73, and an F1 score of 0.67, along with a p-value of 1.815e-4. In the case of 0-100 mM, the performance metrics indicated an accuracy of 0.77, a sensitivity of 0.51, a specificity and precision of 1, and an F1 score of 0.67, with a p-value of 4.257e-06. For the 0-50 mM tests using all attributes, the results included an accuracy of 0.64, a sensitivity of 0.75, a specificity of 0.54, a precision of 0.59, an F1 score of 0.65, and a p-value of 0.01.

When considering only the color attributes, the results for 0-200 mM included an accuracy of 0.94, a sensitivity of 0.88, a specificity and precision of 1, an F1 score of 0.93, and a p-value lower than 2.2e-16. For 0-150 mM, the values were 0.80, 0.71, 0.88, 0.83, 0.77, and a p-value of 9.294e-08. For 0-100 mM, the results were 0.75, 0.48, 1, 1, 0.66, and a p-value of 4.551e-08. In the case of 0-50 mM, the values were 0.56, 0.50, 0.61, 0.52, 0.51, and no significant p-value was observed.

When using only the morphological attributes for 0-200 mM, an accuracy of 0.94, a sensitivity of 0.88, a specificity and precision of 1, an F1 score of 0.88, and a Fisher’s exact test p-value lower than 2e-16 were achieved. In the 0-150 mM group, the metrics were 0.74, 0.86, 0.64, 0.67, 0.75, and the p-value was 7.432e-06. For the 0-100 mM and 0-50 mM groups, the values obtained were 0.74, 0.58, 0.88, 0.82, 0.69, and 1.498e-05, and 0.53, 0.94, 0.19, 0.50, 0.65, and 0.098, respectively.

**Table 7** displays the performance metrics for VISBACK in sets 2 and 4. When considering all attributes in the 0-200 mM concentration range, the metrics included an accuracy of 0.71, a sensitivity of 0.90, a specificity of 0.52, a precision of 0.66, and an F1 score of 0.76, with a Fisher’s exact test p-value of 3.601e-05. For the 0-150 mM tests, the metrics showed results of 0.73 for accuracy, 0.67 for sensitivity, 0.79 for specificity, 0.73 for precision, and an F1 score of 0.70, with a p-value of 7.798e-05. In the case of 0-100 mM and 0-50 mM, the metrics values were 0.47, 0.56, 0.38, 0.46, and 0.50, and 0.42, 0.63, 0.23, 0.42, and 0.51, respectively. In both cases, the p-values were not significant.

When using only the color attributes, the performance metrics at the 0-200 mM concentrations were as follows: an accuracy of 0.74, a sensitivity of 0.69, a specificity of 0.78, a precision of 0.76, and an F1 score of 0.72. In the 0-150 mM group, the metrics displayed values of 0.73, 0.77, 0.69, 0.68, and 0.78, respectively. For 0-100 mM and 0-50 mM, the metrics indicated 0.49, 0.52, 0.43, 0.48, and 0.51, and 0.60, 0.83, 0.40, 0.55, and 0.66, respectively. Notably, only 0-200 mM and 0-150 mM presented significant p-values (p< 0.01). The performance metrics when considering only the morphological attributes exhibited an accuracy of 0.82, a sensitivity of 0.64, a specificity and precision of 1, and an F1 score of 0.78. In the 0-150 mM group, the values were 0.60, 0.63, 0.80, 0.61, and 0.46. For 0-100 mM, the metrics indicated values of 0.61, 0.36, 0.36, 0.86, and 0.47, and for 0-50 mM, the values were 0.44, 0.44, 0.45, 0.41, and 0.42. Only the 0-200 mM group showed a significant p-value (p< 0.01).

### 3.6 K-fold validation portal performance among groups

**Figure 6:**
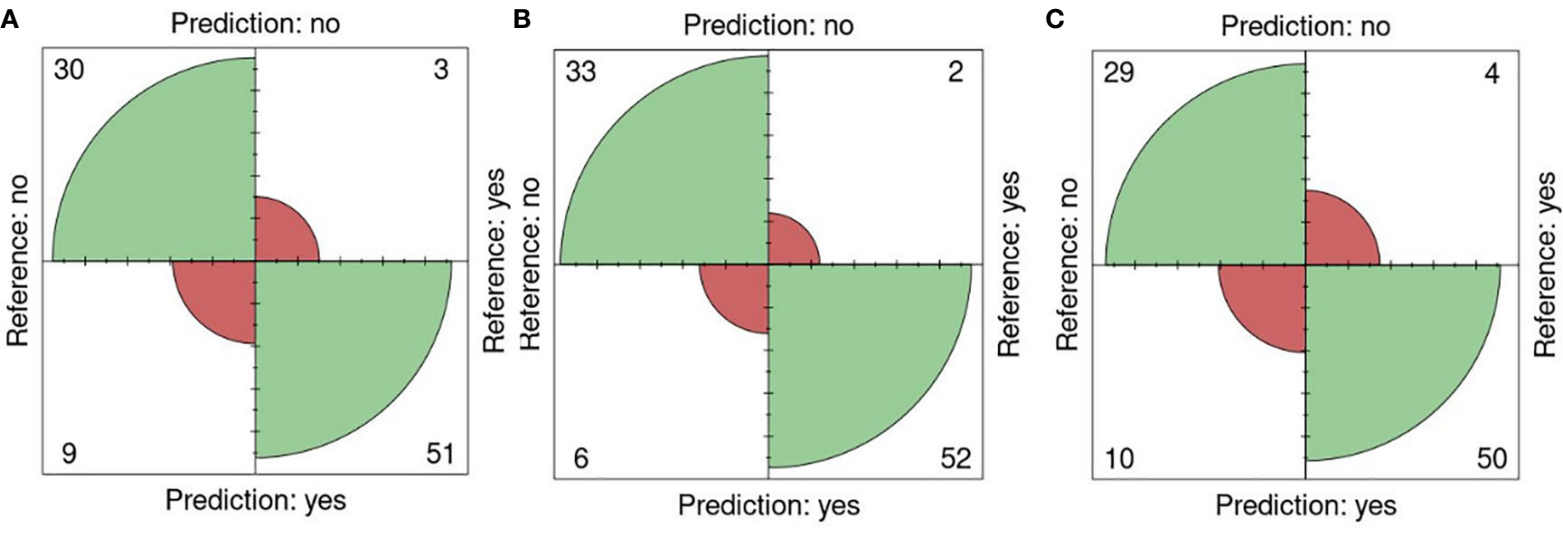
K-fold validation confusion matrices. Reference (real) versus prediction plots among groups using one training plate for each condition (salt/nonsalt) with a k=10 for 0 and 200mM. **(A)** All attributes, **(B)** Color attributes, **(C)** Morphological attributes.

The performance of the portal and the type of sensor was performed using the k-fold validation technique which is normally used to test machine learning algorithms with a k equal to 10 (Sakeef et al., 2023), on fluorescent images (FLUO). The salt concentration chosen was 0-200 mM since it is present in all the sets. Out of 93 plates, 30 were well classified as non-salt and 51 as salt against 9 misclassified as salt and 3 as non-salt for all attributes (**Figure 6A**). Using only the color attributes, 33 and 52 were well classified as non-salt and salt and 6 and 2 misclassified as salt and non-salt (**Figure 6B**). In the case of only morphological attributes, 29 and 50 were well classified against 10 and 4 respectively (**Figure 6C**).

An accuracy of 0.87 was attained using all attributes, accompanied by a sensitivity of 0.94, a specificity of 0.76, a precision of 0.85, and an F1 score of 0.89. When exclusively employing color attributes, an accuracy of 0.91 was achieved, along with a sensitivity of 0.96, a specificity of 0.84, a precision of 0.90, and an F1 score of 0.93. In the case of using only morphological attributes, results included an accuracy of 0.84, a sensitivity of 0.90, a specificity of 0.74, a precision of 0.83, and an F1 score of 0.88. Significance (p< 0.01) in all cases was shown using Fisher’s exact test (**Table 8**).

**Table 8:** Performance descriptors of k-fold validation tests among groups using 0 and 200mM from fluorescent images (FLUO).

Attributes	Accuracy Equation 1	Sensitivity Equation 2	Specificity Equation 3	Precision Equation 4	F1 score Equation 5	p-value*
All	0.87	0.94	0.76	0.85	0.89	3.339e-13
Color	0.91	0.96	0.84	0.90	0.93	< 2.2e-16
Morpho	0.84	0.93	0.74	0.83	0.88	1.289e-11

* Fisher’s exact test.

### 3.7 Alternative applications of SeedML

**Figure 7:**
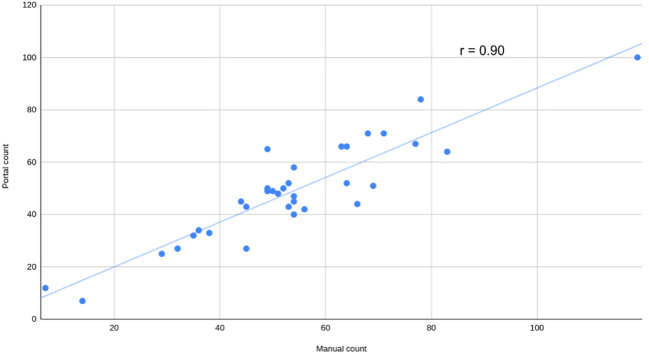
Manual versus automatic count of pods per plant. Manual count of pods versus automatically calculated by the portal using visible light side view images from a mix of Camelina plants grown in salt and non-salt condition at 52 days after sowing. The Pearson correlation coefficient (r) is shown.

To assess the usability of the portal for working with various types of data, a series of side-view images of Camelina plants were captured and analyzed using this portal. The parameters for quantifying and qualifying pods per plant were adjusted through the user interface section “seed detection setup”. Manual counting was also completed to evaluate performance. The strength of the relationship was assessed using the Pearson coefficient (r=0.90), revealing a strong positive correlation (**Figure 7**).

## 4 Discussion

The morpho-colorimetric seed features using the fluorescent light images displayed a greater sensitivity to salt than the visible light images (**Figures 2**, **3**). In fact, the area-related features showed higher values in the fluorescent images under salt conditions as well as the lower, median and higher quartiles of the red intensity value. This may be explained by the fluorescence emission intensity which increases with the increase in concentration of salt (Adenier et al., 1998; Sharma et al., 2018). A variation in the morpho-colorimetric seed features was also observed among the sets. This variation may be attributed to differences in the chemical composition of seed oil (Dogruer et al., 2021), which could be influenced by variations in growing conditions, including watering regimes. It has been shown that seed oil content can change in response to factors such as nitrogen fertilizer, suggesting that soil content including the prevalence of salts may play a key role in seed oil composition (Li et al., 2017).

The conversion from pixels to the metric system is important not only for the purpose of comparing and sharing information, as it does not depend on the image, but also for validating the results of seed detection. This feature is included in the portal. We used the measurements of the plate in both cameras to calculate the conversion and we compared seeds manually measured using a ruler (**Supplementary Figure 1**). Our manual observation and pixel-converted calculation both yielded a length of 2 mm, which aligns with the measurements reported by Francis and Warwick (2009). Additionally, the portal calculated a width of 1 mm, half of the length, in line with the findings of Fleenor (2011). An amount of 1000 seeds weighs between 0.8 to 2.0 g (Ehrensing et al., 2008), meaning that the number of seeds expected in 0.1 g is in the range of 50 to 125 seeds which has been corroborated in our analysis with an average of 92, 84 and 95 normally distributed between 60-140.

The machine learning algorithms evaluated on the classification of individual seeds were taken from the WEKA package (Frank et al., 2016), namely ZeroR, NaiveBayes, MultilayerPerceptron, SMO, IBk, Kstar, LWL, DecisionStump, HoeffdingTree, J48, LMT, RandomForest, RandomTree and REPTree (**Figure 1**, **Table 4**). All of them show an accuracy equal or greater than 70% except for the ZeroR which showed an accuracy of 52% (**Figure 4**). For this reason, the ZeroR algorithm was not implemented in the portal since it did not significantly contribute to the classification process.

The consensus achieved by the machine learning algorithms analyzing morpho-colorimetric features in the image analysis process, in conjunction with the universally accessible user-friendly web interface and a wide range of customizable parameters, endows the portal with exceptional performance. The outputs may be tailored to accommodate various types of images, to inform on a wide range of data sets. Most of the analyses were conducted using a different plate for training in each group or set, as it represents the minimum information that can be provided. However, a three-plate training approach was implemented to uphold this principle. The best performance, achieved using the one-plate training method, was observed in the case of the fluorescent light images, with scores of 90% or higher in all five effectiveness metrics. This was followed by the visible light back images and then by the visible light top images. In the case of three-plate training, almost 100% classification performance was obtained in the five metrics (**Figure 5**, **Table 5**). This demonstrates the robustness of the algorithms implemented in the portal, as well as the effect of salt on fluorescent light reflectance (Adenier et al., 1998; Sharma et al., 2018). Furthermore, utilizing color attributes alone resulted in an overperformance compared to using only morphological attributes (**Table 5**).

The reduction in salt concentration resulted in a decrease in the effectiveness of the classification. This effect was observed in two sets of fluorescent light images where lower concentrations were available (**Table 6**). This finding supports the influence of salt on fluorescent reflectance and may indicate a lower concentration of salt within the seeds when grown in less saline soils. In 0-200 mM, the F1 score is 0.95 compared to 0.65 in 0-50mM. This may represent a correlation between the seed salt content and the fluorescent seed reflectance.

The k-fold validation is a widely used method to estimate the performance of machine learning algorithms on many performance indicators, in this case, accuracy, sensitivity, specificity, precision and F1 score (Refaeilzadeh et al., 2009). A k value equal to 10 was used since it is the most acceptable value for testing these kinds of algorithms (Refaeilzadeh et al., 2009; Sakeef et al., 2023). The 0-200 mM concentrations were selected from sets 1 to 6 (**Figure 6**, **Table 8**). This allows us to test the performance of the prediction process among groups growing in different conditions using fluorescent light images. Surprisingly, an accuracy of 0.87 and 0.91 was achieved with all and color attributes only and a sensitivity of 0.94 and 0.96 respectively even though the fluorescent reflectance is also affected by the oil composition which is affected by the growing conditions (Boschi et al., 2011; Li et al., 2017; Cober and Malcolm, 2019; Dogruer et al., 2021).

The SeedML portal offers a versatile solution for addressing various phenotypic questions using plant images. As an illustrative case, this research showcases the automated counting of pods in side-view images of Camelina. This data is crucial for evaluating yield production and would otherwise demand significant human resources and time if handled manually. In this case, achieving the objective was accomplished by simply adjusting parameters through the user interface. A high Pearson correlation coefficient (r = 0.90) was obtained, indicating the effectiveness of this analysis. It should be noted that this was just one illustrative example and the SeedML portal can be used to perform a wide range of image-based phenotyping analyses.

In this study, the capability of combining fluorescent and visible light images with image analysis and machine learning algorithms to assess the color-morphological characteristics of Camelina seeds to predict the soil’s salinity status has been demonstrated. An easy to navigate portal was devised and designed to be accessible to individuals with minimal computer skills and compatible with any device, including smartphones. The utility of the portal in addressing other phenomics analyses along with its implications in oil assessment and quality control have been illustrated. The findings of this research may positively inform related studies in the context of agricultural innovation and related fields such as animal feed production, in response to climate change. SeedML may further aid in the development and implementation of new quality control tools within the agri-food industry, enhancing productivity and sustainability in the manufacturing process.

## Data availability statement

The raw data supporting the conclusions of this article will be made available by the authors, without undue reservation.

## Author contributions

EV: Conceptualization, Data curation, Formal analysis, Investigation, Methodology, Software, Validation, Writing – original draft, Writing – review & editing. ML: Conceptualization, Investigation, Methodology, Validation, Writing – original draft, Writing – review & editing. JA: Conceptualization, Investigation, Writing – review & editing. TB: Conceptualization, Funding acquisition, Resources, Supervision, Writing – review & editing.
