# Supplementary material for: Integrated web portal for non-destructive salt sensitivity detection of Camelina sativa seeds using fluorescent and visible light images coupled with machine learning algorithms
Source: Front Plant Sci. 2024 Jan 11;14:1303429. doi: 10.3389/fpls.2023.1303429 (PMC10808381; doi:10.3389/fpls.2023.1303429)
Supplement: Supplementary file 1 [file DataSheet_1.pdf]

A

Setting scale pixels/cm (fluorescent)

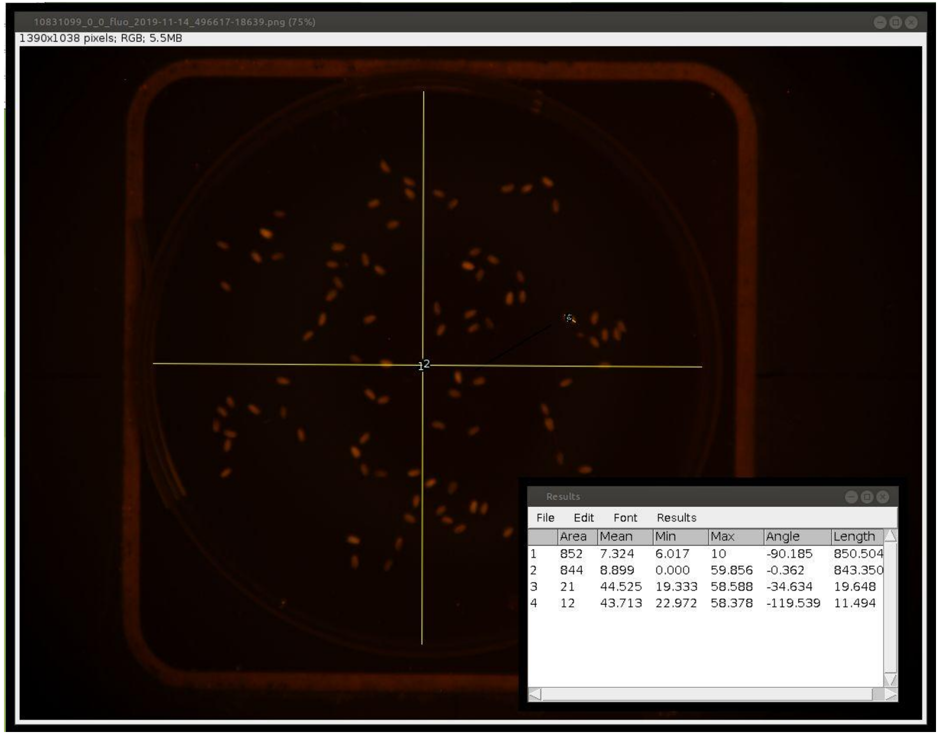

	Area	Mean	Min	Max	Angle	Length
1	852	7.324	6.017	10	-90.185	850.504
2	844	8.899	0.000	59.856	-0.362	843.350
3	21	44.525	19.333	58.588	-34.634	19.648
4	12	43.713	22.972	58.378	-119.539	11.494

- Using inside diameter 8.5 cm
- (850 + 843)px / 2 = 846.5
- 846.5 px / 8.5 cm = 99.58 px / cm

- Average from process img
- 9.76 px major axis (long/2)
- 5.17 px minor axis (wide/2)
- 1.95 mm x 1.03 mm
- Agreed with visible

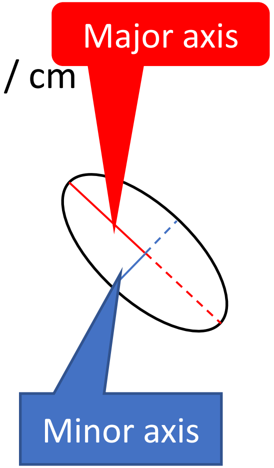

B

Setting scale pixels/cm (visible back light)

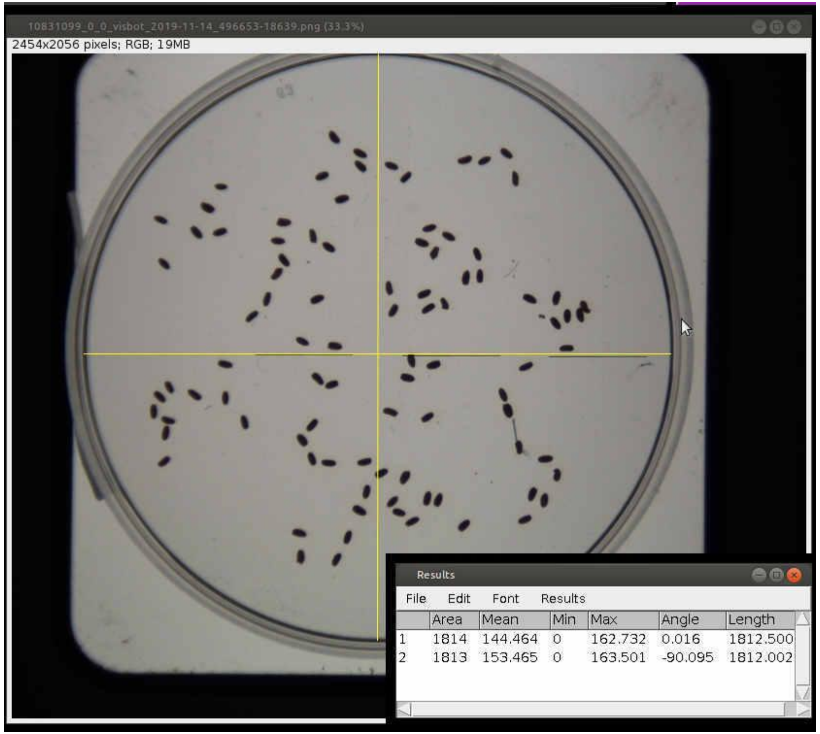

- Using inside diameter 8.5 cm
- 1812 px both measurements
- 1812 px / 8.5 cm = 213.17 px / cm

- Average from process img
- 21.75 px major axis (long/2)
- 10.83 px minor axis (wide/2)
- 2 mm x 1 mm
- Agreed with fluorescent

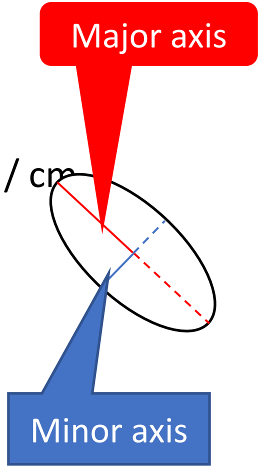

c

Camelina seed size (manual validation)

- According to Fleenor, R . (2011) less 1/16 inch long and about half as wide. (1.59 x 0.80 mm)
- According to Francis et Warwick (2009) between 2 and 3 mm long
- Our manual measurements shows about 2 mm. This agreed with our computational calculation in both cameras.

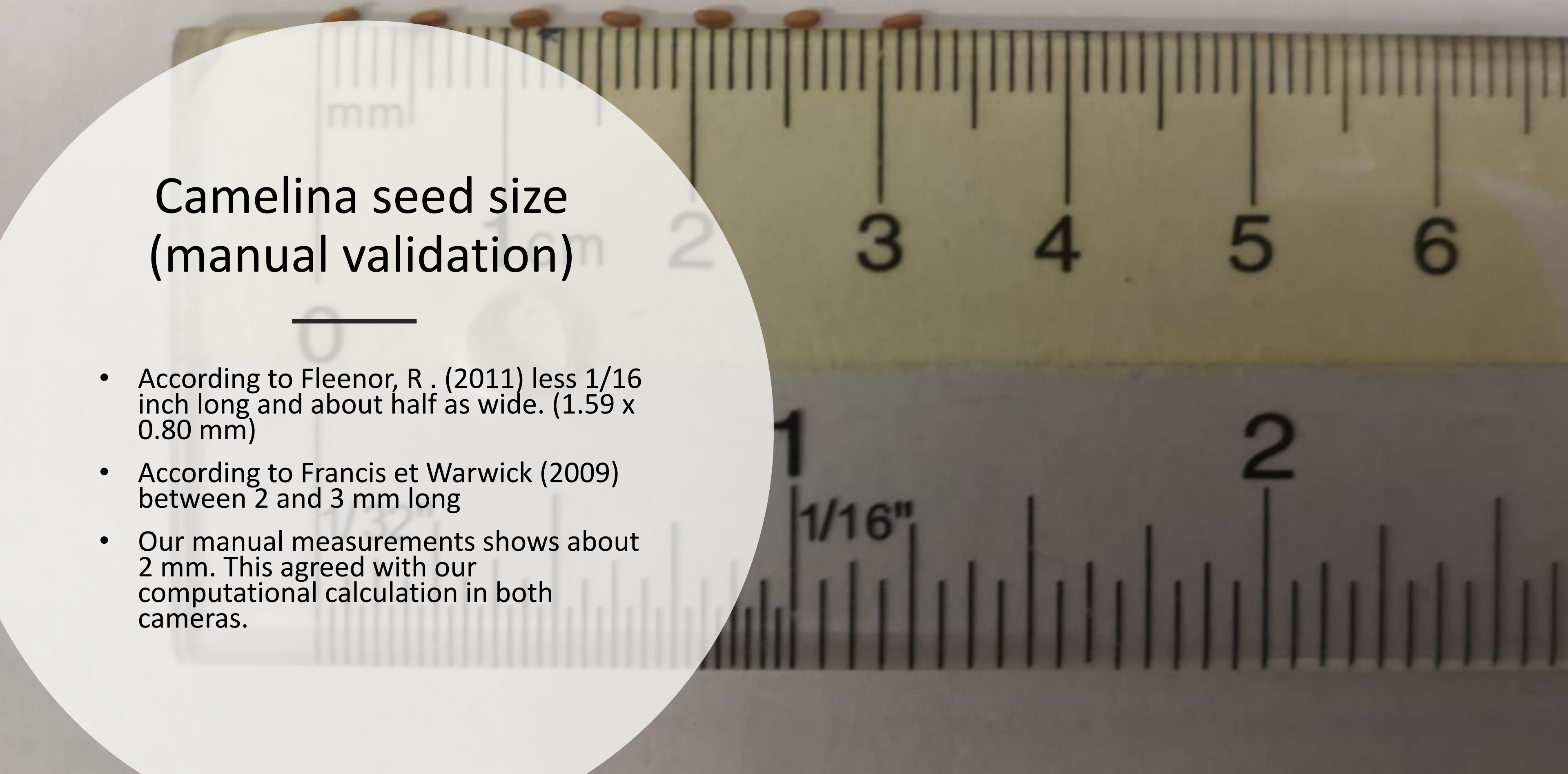

D

Number of seeds counted automatically through the portal.

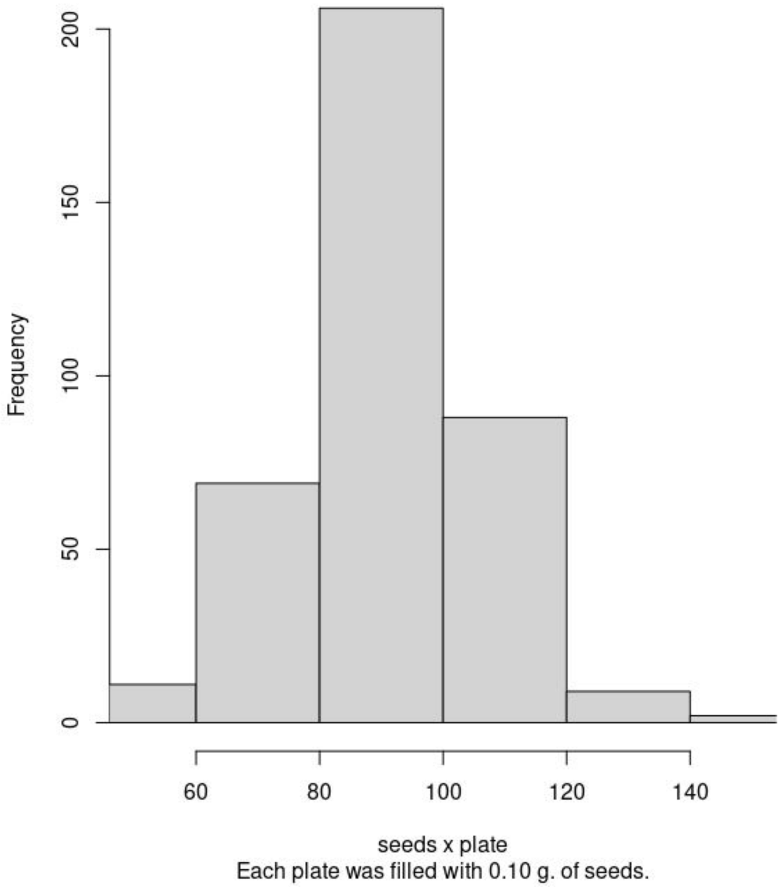


```
Fluorescent light camera (FLUO)
95 percent confidence interval:
 89.52574 95.20708
sample estimates:
mean of x
 92.36641

Visible top light camera (VISFRONT)
95 percent confidence interval:
 80.45794 88.25962
sample estimates:
mean of x
 84.35878

Visible back light camera (VISBACK)
95 percent confidence interval:
 92.54023 99.30710
sample estimates:
mean of x
 95.92366
```
