# Supplementary material for: Integrated web portal for non-destructive salt sensitivity detection of Camelina sativa seeds using fluorescent and visible light images coupled with machine learning algorithms
Source: Front Plant Sci. 2024 Jan 11;14:1303429. doi: 10.3389/fpls.2023.1303429 (PMC10808381; doi:10.3389/fpls.2023.1303429)
Supplement: Supplementary file 2 [file DataSheet_2.pdf]

Testing Lists Inside Groups with One Training Plate per Group

Camera	Attribute	Set	Spec Mm	1	2	3
FLUO	All	1	0-200	1	2	3
			0-250	4	5	6
		2	0-50	7	8	9
			0-100	10	11	12
			0-150	13	14	15
			0-200	16	17	18
		3	0-200	19	20	21
		4	0-50	22	23	24
			0-100	25	26	27
			0-150	28	29	30
			0-200	31	32	33
		5	0-200	34	35	36
		6	0-200	37	38	39
	Morpho	1	0-200	40	41	42
			0-250	43	44	45
		2	0-50	46	47	48
			0-100	49	50	51
			0-150	52	53	54
			0-200	55	56	57
		3	0-200	58	59	60
		4	0-50	61	62	63
			0-100	64	65	66
			0-150	67	68	69
			0-200	70	71	72

Camera	Attribute	Set	Spec Mm	1	2	3
		5	0-200	73	74	75
		6	0-200	76	77	78
	Colour	1	0-200	79	80	81
			0-250	82	83	84
		2	0-50	85	86	87
			0-100	88	89	90
			0-150	91	92	93
			0-200	94	95	96
		3	0-200	97	98	99
		4	0-50	100	101	102
			0-100	103	104	105
			0-150	106	107	108
			0-200	109	110	111
		5	0-200	112	113	114
		6	0-200	115	116	117
VISBACK	All	1	0-200	118	119	120
			0-250	121	122	123
		2	0-50	124	125	126
			0-100	127	128	129
			0-150	130	131	132
			0-200	133	134	135
		3	0-200	136	137	138
		4	0-50	139	140	141
			0-100	142	143	144
			0-150	145	146	147
			0-200	148	149	150
		5	0-200	151	152	153
		6	0-200	154	155	156

Camera	Attribute	Set	Spec Mm	1	2	3
	Morpho	1	0-200	157	158	159
			0-250	160	161	162
		2	0-50	163	164	165
			0-100	166	167	168
			0-150	169	170	171
			0-200	172	173	174
		3	0-200	175	176	177
		4	0-50	178	179	180
			0-100	181	182	183
			0-150	184	185	186
			0-200	187	188	189
		5	0-200	190	191	192
		6	0-200	193	194	195
	Colour	1	0-200	196	197	198
			0-250	199	200	201
		2	0-50	202	203	204
			0-100	205	206	207
			0-150	208	209	210
			0-200	211	212	213
		3	0-200	214	215	216
		4	0-50	217	218	219
			0-100	220	221	222
			0-150	223	224	225
			0-200	226	227	228
		5	0-200	229	230	231
		6	0-200	232	233	234
VISFRONT	All	1	0-200	235	236	237
			0-250	238	239	240

Camera	Attribute	Set	Spec Mm	1	2	3
		2	0-50	241	242	243
			0-100	244	245	246
			0-150	247	248	249
			0-200	250	251	252
		3	0-200	253	254	255
		4	0-50	256	257	258
			0-100	259	260	261
			0-150	262	263	264
			0-200	265	266	267
		5	0-200	268	269	270
		6	0-200	271	272	273
	Morpho	1	0-200	274	275	276
			0-250	277	278	279
		2	0-50	280	281	282
			0-100	283	284	285
			0-150	286	287	288
			0-200	289	290	291
		3	0-200	292	293	294
		4	0-50	295	296	297
			0-100	298	299	300
			0-150	301	302	303
			0-200	304	305	306
		5	0-200	307	308	309
		6	0-200	310	311	312
	Colour	1	0-200	313	314	315
			0-250	316	317	318
		2	0-50	319	320	321
			0-100	322	323	324

Camera	Attribute	Set	Spec Mm	1	2	3
			0-150	325	326	327
			0-200	328	329	330
		3	0-200	331	332	333
		4	0-50	334	335	336
			0-100	337	338	339
			0-150	340	341	342
			0-200	343	344	345
		5	0-200	346	347	348
		6	0-200	349	350	351
